# Orexin‐A mediates glioblastoma proliferation inhibition by increasing ferroptosis triggered by unstable iron pools and GPX4 depletion

**DOI:** 10.1111/jcmm.18318

**Published:** 2024-04-29

**Authors:** Rengzheng Huan, Jiqin Zhang, Jianhe Yue, Sha Yang, Guoqiang Han, Yuan Cheng, Ying Tan

**Affiliations:** ^1^ Department of Neurosurgery The Second Affiliated Hospital of Chongqing Medical University Chongqing China; ^2^ Department of Anesthesiology Guizhou Provincial People's Hospital Guiyang China; ^3^ Department of biomedical sciences Medical College of Guizhou University Guiyang China; ^4^ Department of Neurosurgery Guizhou Provincial People's Hospital Guiyang China

**Keywords:** ferroptosis, glioblastoma, orexin‐A

## Abstract

Glioblastoma (GBM) represents a prevalent form of primary malignant tumours in the central nervous system, but the options for effective treatment are extremely limited. Ferroptosis, as the most enriched programmed cell death process in glioma, makes a critical difference in glioma progression. Consequently, inducing ferroptosis has become an appealing strategy for tackling gliomas. Through the utilization of multi‐omics sequencing data analysis, flow cytometry, MDA detection and transmission electron microscopy, the impact of orexin‐A on ferroptosis in GBM was assessed. In this report, we provide the first evidence that orexin‐A exerts inhibitory effects on GBM proliferation via the induction of ferroptosis. This induction is achieved by instigating an unsustainable increase in iron levels and depletion of GPX4. Moreover, the regulation of TFRC, FTH1 and GPX4 expression through the targeting of NFE2L2 appears to be one of the potential mechanisms underlying orexin‐A‐induced ferroptosis.

## 1 INTRODUCTION

Glioblastoma (GBM), as the most lethal subtype of glioma, represents a prevalent form of primary tumours in central nervous system (CNS).^1^ Continuously updated treatment methods including surgical resection, temozolomide (TMZ) chemotherapy, radiation therapy, electric field therapy and immunotherapy have extended the median survival of GBM patients to some extent (the median survival at 14.6 months), but the overall effect is still unsatisfactory.^2^ Therefore, there is an urgent and imperative demand to discover novel therapeutic strategies and antitumour medications to surmount this predicament.

Ferroptosis is a form of regulated cell death (RCD) that is dependent on iron, and is caused by unchecked lipid peroxidation resulting from excessive generation of reactive oxygen species (ROS) and iron overload.^2^, ^3^ Cumulative evidence suggests that ferroptosis makes a critical difference in human cancer, and several drugs have displayed potential against tumours by targeting regulatory molecules involved in ferroptosis.^3^, ^4^ Moreover, it has been reported that GBMs exhibit higher sensitivity to ferroptosis compared to other cancer types.^5^, ^6^ In the context of drug‐resistant GBM, roxadustat has been discovered to promote ferroptosis by activating HIF‐α.^7^ Additionally, Xu et al. demonstrated that sevoflurane can drive ferroptosis in GBM cells.^8^ Hence, it is of utmost importance to thoroughly study the mechanisms of ferroptosis in GBM and identify suitable target drugs to enhance the prognosis of GBM patients.

Orexin‐A, a multifunctional neuropeptide produced by orexin neurons, is an important participant in the pathogenesis of type I narcolepsy, chronic inflammatory neurodegenerative metabolic syndrome, and cancer.^9^, ^10^, ^11^, ^12^, ^13^ Orexin‐A exerts its effects by binding to orexin‐A receptor and activates a variety of downstream signalling pathways involved in anti‐inflammatory, neuroprotective and immune regulation.^14^, ^15^, ^16^, ^17^ Interestingly, a series of researches focused on the interaction of orexin‐A with tumours in recent years discovered that orexin‐A shows an exciting potential in the treatment of certain types of cancer.^15^, ^18^, ^19^, ^20^ Orexin‐A has been reported to play an antitumor role by inducing tumour cell apoptosis and enhancing antitumour immunity, and is considered as a promising antitumor drug.^21^ Furthermore, our previous investigations have revealed the antitumor role of orexin‐A in GBM. Through multiple histological analyses of the transcriptome, proteome and metabolome, we have observed that orexin‐A may potentially target ferroptosis, although the specific mechanism remains unclear.^22^, ^23^

In this study, we assessed the impact of orexin‐A on GBM ferroptosis and delved deeper into the specific mechanism behind orexin‐induced GBM ferroptosis. Our findings indicate that orexin‐A effectively triggered ferroptosis in GBM while impeding their proliferation. Targeting NFE2L2 regulates the expression of TFRC, FTH1 and GPX4, induces the increase of unstable iron and the depletion of GPX4, which is an important mechanism of orexin‐A‐mediated GBM ferroptosis.

## 2 METHOD

### 2.1 Reagents and sources of reagents

Orexin‐A (Cat. No. 1455) was purchased from TOCRIS Bioscience (ENGLAND). Ferrostatin‐1 (HY‐100579) and TBHQ (HY‐100489) were purchased from MCE (CHINA). Antibodies against Ki‐67 (D3B5), TFRC (D7G9X), FTH1 (D1D4) and GAPDH (D16H11) were purchased from Cell Signaling (USA). Antibodies against NFE2L2 (ab62352), GPX4 (ab125066) and β‐actin (ab8226) were purchased from Abcam (ENGLAND).

### 2.2 Cell lines and cell culture conditions

All the cells involved in this experiment were obtained from the Chinese academy of sciences cell bank (Shanghai, China). Human GBM cell lines U251‐MG and U87‐MG were cultured in Dulbeccos modified Eagle's essential medium (DMEM) supplemented with 10% foetal bovine serum (Gibco, USA) and 1% penicillin/streptomycin (Beyotime, China) under 37°C and 5% CO_2_ condition.

### 2.3 CCK8 assay

Cell proliferation capacity was carried out with CCK‐8 Kit (Beyotime, China). The collected cells were fully resuspended and seeded in 96‐well plates at 5 × 10^3^ cells per well. Cells were cultured overnight and exposed to orexin‐A (0.1 μM). In the rescue experiments, Fer‐1 (5 μM) were added in cells 1 h before orexin‐A treatment. The absorbance of different treatment groups was detected by microplate reader (BioTek, USA).

### 2.4 Colony formation assay

Cells were seeded in six‐well plates at a concentration of 500 cells per well and cultured for 2 weeks in the presence of various concentrations of orexin‐A (0.1 μM). In the rescue experiments, Fer‐1 (5 μM) were added in cells 1 h before orexin‐A treatment. Colonies were fixed with 4% paraformaldehyde (Biosharp, Anhui, China) and stained with 0.1% crystal violet (G1014, Servicebio, Wuhan). Then, effective colonies were screened and counted.

### 2.5 Measurement of intracellular glutathione (GSH) levels

GSH levels were determined by relevant detection kits (BC1175, Solarbio, China) as per the provided instructions. GBM tissues or cells treated with different interventions were collected, and the relevant detection reagents were added, ground thoroughly on ice, and centrifuged at 10000×*g* for 10 min at 4°C, and the supernatant was removed for determination of GSH.

### 2.6 Measurement of malondialdehyde (MDA)

MDA levels were assessed using the appropriate detection kits (S0131M, Beyotime, China) as per the provided instructions. GBM tissues or cells subjected to different interventions were collected and homogenized on ice with lysate for supernatant collection. The obtained supernatant was then combined with the assay solution in a 1:2 ratio, followed by incubation at 100°C for 15 min. The optical density (OD) was subsequently determined at 530 nm using a microplate reader.

### 2.7 Determination of intracellular ROS levels

The level of intracellular ROS was determined by relevant detection kits (Thermo Fisher Scientific, USA) as per the provided instructions. Cells were seeded in six‐well plates and cultured overnight. Then, cells were exposed to different concentrations of orexin‐A for 24 h and incubated with 10 μM DCFH‐DA for 30 min in the dark. Fluorescence microscopy (Olympus, Japan) and flow cytometry (BD Biosciences; San Jose, CA, USA) were used to determine ROS levels.

### 2.8 Iron assay

The cell iron determination kit (BC5315) of solarbio and tissue iron determination kit (A039‐2) of Nanjing Jiancheng Bioengineering Institute were used to determine the iron content of cells and tissues, respectively. Glioma cells or tissues treated with different interventions were collected and ground on ice. The concentration of iron was assessed by measuring the absorbance of the sample at 520 nm.

### 2.9 Transmission electron microscopy

GBM cells were collected and initially treated with a 3% glutaraldehyde solution. Subsequently, the cells were postfixed using a 1% osmium tetroxide solution. Afterward, a series of acetone dehydration steps were carried out, followed by infiltration with Epon 812 and subsequent embedding. The resulting semithin sections were stained using methylene blue, while ultrathin sections were sliced using a diamond knife and then stained with uranyl acetate and lead citrate. Finally, the sections were examined using a JEM‐1400‐FLASH Transmission Electron Microscope.

### 2.10 Sequencing analysis

The transcriptome, proteomics and metabolomics data were obtained by sequencing analysis for three repeats of orexin‐A‐treated GBM cells and three repeats of untreated GBM cells. The limma package (version 3.44.3) was employed to analyse the differences between the different treatment groups in transcriptome, proteome and metabolome data (|log2fold change (FC)|>0.5, *p*‐value < 0.05). All data in the three groups were included in the combined KEGG analysis to investigate the transcriptome‐proteome‐metabolome interactions. All common enriched pathways of DEGs, DEPs and DEMs were tested by hypergeometric distribution test (*p*‐value < 0.05).

### 2.11 Quantitative reverse transcription–polymerase chain reaction

After being treated with 0.1 μM orexin‐A for 24 h, total RNA was extracted and reverse‐transcribed into cDNA using a cDNA transcription kit. Quantitative RT‐PCR was performed by the real‐time fluorescence quantitative PCR instrument (CFXOpus 384, Bio‐Rad, USA). The relative expression levels of target genes were normalized to GAPDH via the 2 − ΔΔCt method. The primer distribution is shown in Table S1.

### 2.12 Western blot

Lysates containing a mixture of protease and phosphatase inhibitors were added to the cell samples and lysed by sonication on ice. SDS buffer was added to the lysate and incubated at 100°C in a metal bath for 15 min. Lysates were electrophoresed in corresponding SDS‐PAGE gels, and proteins were separated and transferred to polyvinylidene difluoride membranes (Milipore, USA). Antigen–antibody reactions were performed with the corresponding antibodies under suitable conditions. Protein expression levels were measured using a hypersensitive chemiluminescence kit (American Life Sciences) and normalized using GAPDH.

### 2.13 Immunohistochemistry

Mice tumour tissues were collected and fixed with 4% paraformaldehyde solution. Tissue specimens were embedded in paraffin and sectioned. Immunohistochemical staining of tissue sections was performed with relevant antibodies according to the protocol of the immunohistochemical kit. Five regions were randomly selected from each group and photographed under a microscope. (Olympus, Japan).

### 2.14 Immunofluorescence

Cells were seeded on cell crawl sheet and cultured overnight and then treated with orexin‐A (0.1 μM) for 24 h. After fixation with paraformaldehyde, membrane rupture with triton and sealing with serum, cell slides were incubated with Ki‐67 and TFRC primary antibodies overnight at 4°C. The cell slides were then washed three times with PBS and incubated with the corresponding fluorescent secondary antibody (Molecular Probes, USA) at room temperature in the dark for 1 h. Finally, the cell slides were incubated with DAPI in the dark for 10 min. Five regions were randomly selected from each group and photographed under laser‐scanning confocal microscopy (Olympus, Japan).

### 2.15 Mouse xenografts

The animal study was reviewed and approved by The Institutional Animal Care and Use Committee of Guizhou Province People's Hospital (Guizhou, China). In the experiment of evaluating the effect of orexin‐A on GBM, subcutaneous injection of GBM cells was performed in 6‐week‐old male nude mice, specifically in the proximal region of the right thigh, to establish a xenograft tumour model. The established xenograft tumour model mice were randomly divided into two groups. Orexin‐A (0.1 mg/kg/bw) and double distilled water were respectively administered 1 week after modelling and continued for 2 weeks. Tumour size was recorded every 2 days from the day of administration. In the experiment of evaluating the effect of Ferrostatin‐1 on ferroptosis of glioma mediated by orexin‐A, the xenograft tumour model mice were constructed according to the above methods and randomly divided into three groups with five mice in each group. Double distilled water, orexin‐A (0.1 mg/kg/bw) and orexin‐A (0.1 mg/kg/bw) + Ferrostatin‐1 (1 mg/kg/bw) were, respectively, administered 1 week after modelling and continued for 2 weeks. Tumour size was recorded every 2 days from the day of administration. In the experiment of TBHQ activating NFE2L2, the xenograft tumour model mice were constructed according to the above methods and randomly divided into three groups with five mice in each group. Double distilled water, orexin‐A (0.1 mg/kg/bw) and orexin‐A (0.1 mg/kg/bw) + TBHQ (20 mg/kg/bw) were, respectively, administered 1 week after modelling and continued for 2 weeks. On the next day of the last treatment, all mice were euthanized by cervical dislocation and tumour tissues were excised for immunohistochemical analysis.

### 2.16 Statistical analysis

The comparison between the two groups was examined for statistical significance using the Student's *t*‐test. One‐way ANOVA were conducted to compare the differences among multiple groups. The data are reported as the mean ± standard error of the mean. Results were deemed statistically significant at *p* < 0.05. GraphPad Prism (version 9.0) software was utilized for all analyses.

## 3 RESULTS

### 3.1 Orexin‐A inhibits the proliferation of GBM in vitro and in vivo

**FIGURE 1:**
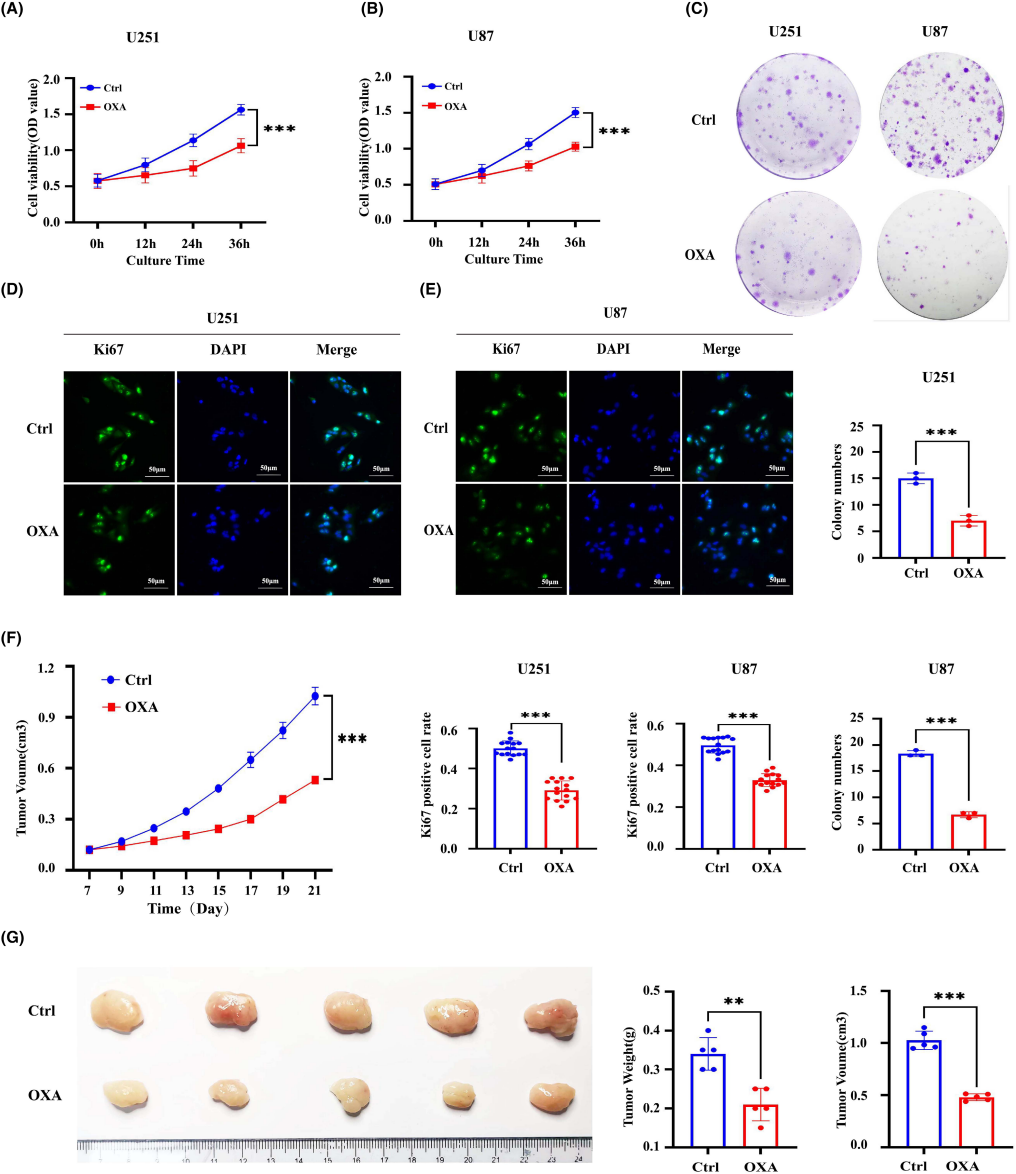
Orexin‐A inhibits glioblastoma proliferation in vivo and in vitro. (A–D) The proliferation of U251 and U87 cells was observed by CCK‐8 method (A and B) colony formation method (C) and Ki67 immunofluorescence method (D and E) after being treated with 0.1 μM orexin‐A for 24 h. Ctrl: treated with double distilled water; OXA: treated with orexin‐A (0.1 μM). (F) Subcutaneous tumour volume curve (*n* = 3). (G) Images of tumours in each group (*n* = 5). Scale bars: 50 μm (400×). Ctrl: treated with double distilled water; OXA: treated with orexin‐A (0.1 mg/kg/bw; **p < 0.01, ***p < 0.001).

To evaluate the effect of orexin‐A on GBM in vitro, we determined the proliferative capacity of GBM cells by CCK8, colony formation and Ki67 immunofluorescence. As shown in Figure 1A,B, CCK8 assay revealed that the OD value of U251 and U87 cells in orexin‐A group was significantly lower than those in the control group. Additionally, treatment with orexin‐A led to a reduction in colony formation in U251 and U87 cells. (Figure 1C) Furthermore, Ki67 immunofluorescence assay demonstrated a significant decrease in the proportion of Ki67‐positive cells in U87 and U251 cells treated with orexin‐A compared to control (Figure 1C,D). These findings provide evidence to support the inhibitory effect of orexin‐A on GBM proliferation in vitro. Moreover, to assess the antitumor capabilities of orexin‐A in vivo, the mouse GBM xenotransplantation model was established by subcutaneous injection of U251 cells. Consistently, in Figure 1F,G, it is clearly evident that tumours in nude mice exposed to orexin‐A displayed markedly reduced weight and diminished volume, thus illustrating the effective inhibition of GBM cell growth by orexin‐A in an in vivo setting.

### 3.2 Ferroptosis is a potential mechanism of orexin‐A inhibition of glioma proliferation

**FIGURE 2:**
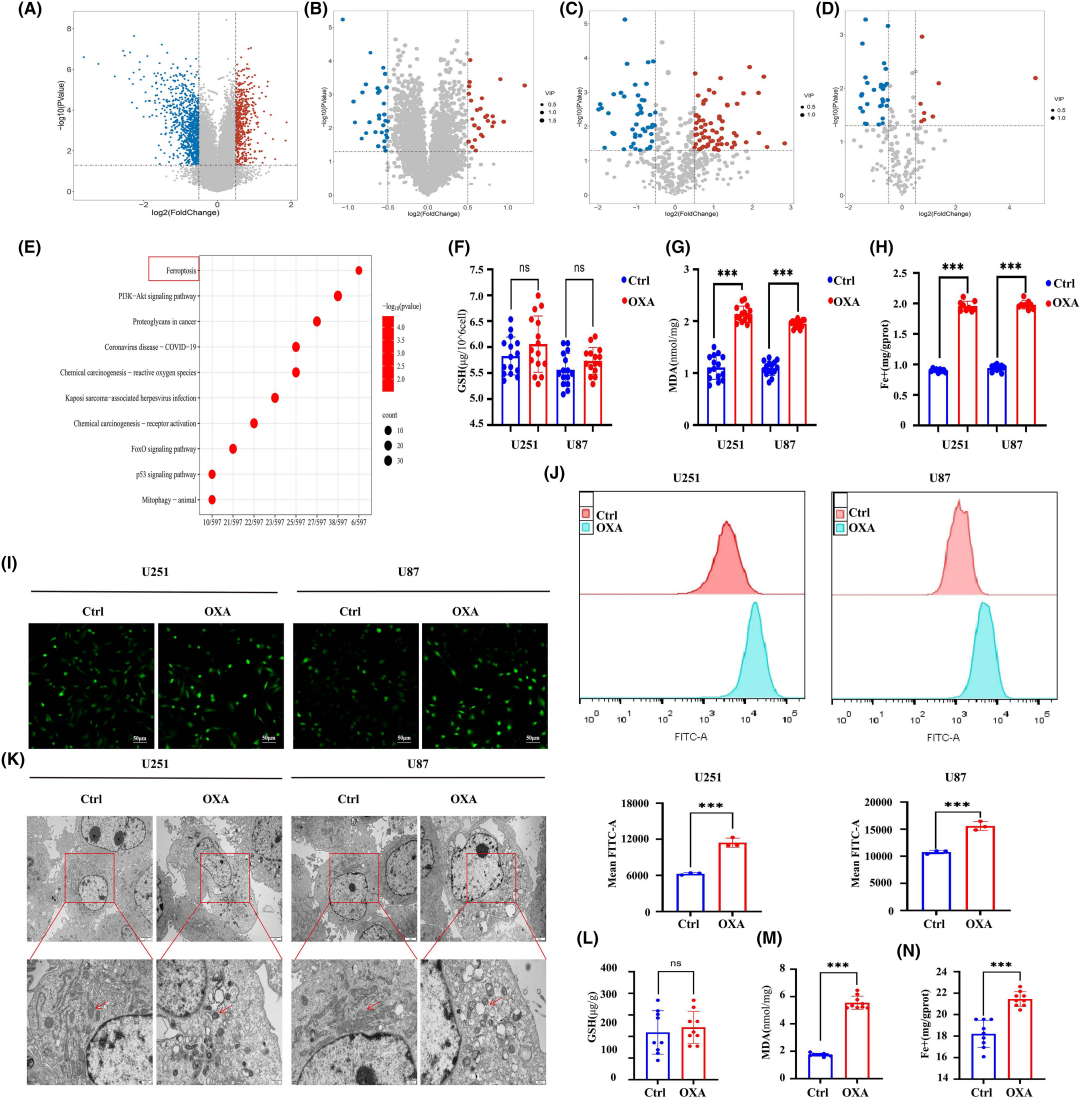
Orexin‐A induces ferroptosis in glioblastoma in vitro and in vivo. (A) Volcano plots of differentially expressed genes (DEGs) between case and control samples with |log2fold change (FC)|>0.5 and *p*‐value < 0.05. (B) Volcano plots of differentially expressed proteins (DEPs) with |log2FC|>0.5, *p*‐value < 0.05 and VIP >1. (C) Volcano plot of differentially expressed metabolites (DEMs) with |log2FC|>0.5, *p*‐value < 0.05 and VIP >1 in positive mode. (D) Volcano plot of DEMs with |log2FC|>0.5, *p*‐value < 0.05 in negative mode. (E) Bubble chart of the 11 most activated KEGG pathways of the DEGs, DEPs and DEMs. (F) Intracellular GSH levels in U251 and U87 cells with or without 0.1 μM orexin‐A treatment for 24 h (*n* = 9). (G) Malondialdehyde (MDA) levels in U251 and U87 cells with or without 0.1 μM orexin‐A treatment for 24 h (*n* = 9). (H) The cellular iron concentration in U251 or U87 cells with 0.1 μM orexin‐A treatment for 24 h. (I) Representative fluorescent images of ROS inU251 or U87 cells with or without 0.1 μM orexin‐A treatment for 24 h. Scale bars: 50 μm (400×). (J) Representative flow cytometry analysis ROS in U251 or U87 cells with or without 0.1 μM orexin‐A treatment for 24 h. (K) Representative cell and mitochondrial ultrastructural images of U251 or U87 cells with or without 0.1 μM orexin‐A treatment for 24 h. Ctrl: treated with double distilled water; OXA: treated with orexin‐A (0.1 μM). (L) GSH levels in Xenograft glioma treated with different reagents. (M) Malondialdehyde (MDA) levels in Xenograft glioma treated with different reagents. (N) The tissue iron concentration in xenograft glioma treated with different reagents. Ctrl: treated with double distilled water; OXA: treated with orexin‐A (0.1 mg/kg/bw; ***p < 0.001).

A total of 1572 DEGs, 52 DEPs and 116 DEMs were identified in our previous studies (Figure 2A–D).^22^ The result of KEGG enrichment analysis for transcriptome‐proteome‐metabolome combination showed that 11 common pathways were significantly enriched by DEGs, DEPs and DEMs, and ferroptosis is one of them. (Figure 2E). Interestingly, previous studies have found that ferroptosis is the most commonly observed mode of programmed cell death (PCD) in glioma.^24^ Therefore, ferroptosis may be a potential mechanism of orexin‐A‐induced GBM inhibitory.

To investigate the ability of orexin‐A to trigger ferroptosis in GBM, we examined the impact of orexin‐A on indicators associated with ferroptosis in vivo and in vitro. In vitro, we observed significant increases in MDA and iron levels in U251 and U87 cells exposed to orexin‐A, but no significant difference in GSH levels. (Figure 2F–H). Moreover, orexin‐A also led to a notable rise in the mean fluorescence intensities of FTIC (Figure 2I,J), indicating a substantial elevation in intracellular ROS levels in U251 and U87 cells. Transmission electronic microscopy was employed to validate the ferroptosis effects induced by orexin‐A, revealing vesicle formation, mitochondrial shrinkage and even the vanishing of the mitochondrial crest in U251 and U87 cells. In vivo investigations further confirmed that orexin‐A caused significant alterations in ferroptosis‐related markers, including increased MDA and iron concentrations (Figure 2L–N). Therefore, both in vitro and vivo, orexin‐A has demonstrated its potential to provoke ferroptosis in GBM.

**FIGURE 3:**
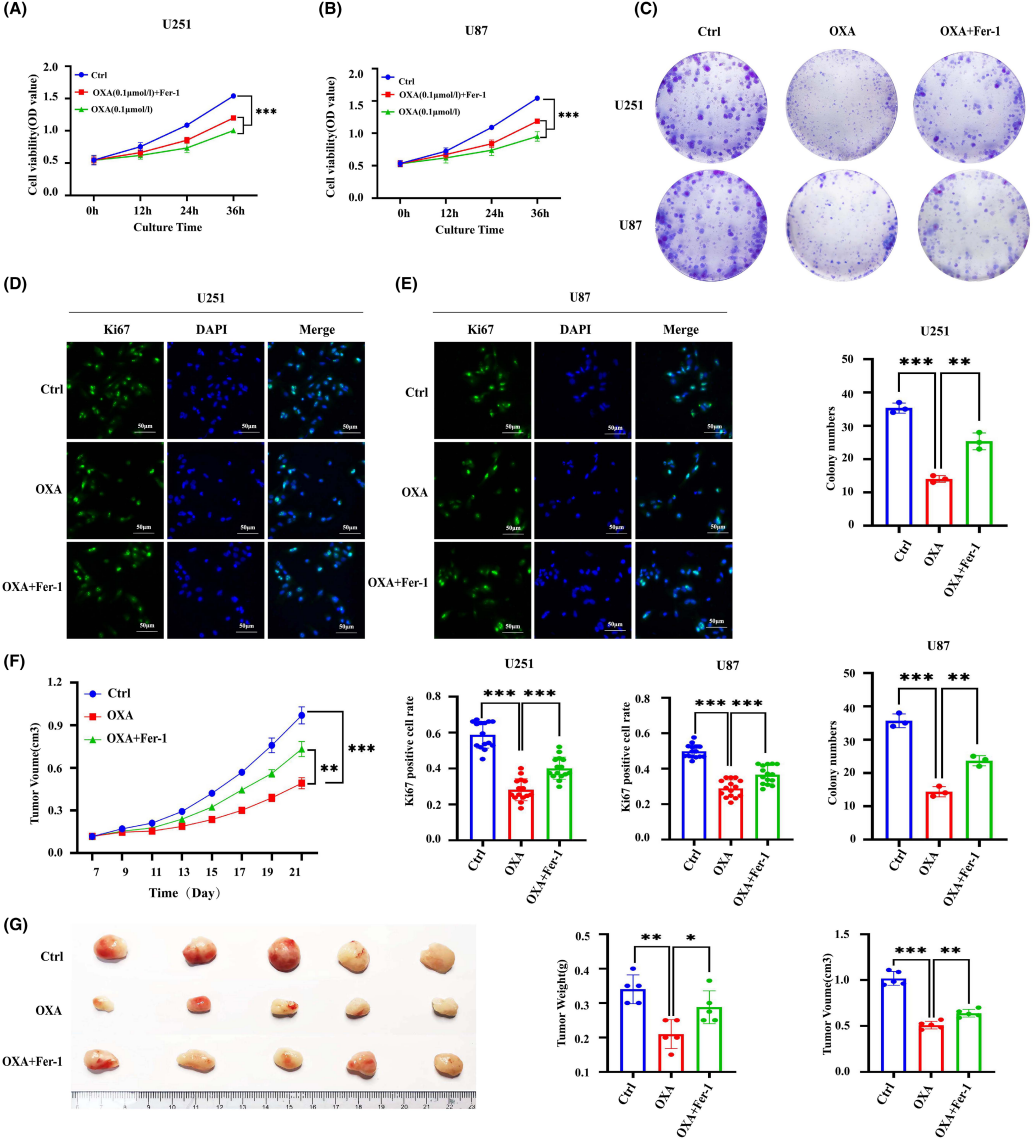
Ferristatin‐1 reverses orexin‐A‐induced inhibition of glioblastoma proliferation. (A–D) Cell proliferation was assessed in U251 and U87 by CCK‐8 assay. (A and B) Colony formation assay (C) and Ki67 immunofluorescence. (D and E) Scale bars: 50 μm (400×). Ctrl: treated with double distilled water; OXA: treated with orexin‐A (0.1 μM); OXA + Fer‐1: treated with orexin‐A (0.1 μM) + ferrostatin‐1 (5 μM). (F) Subcutaneous tumour volume curve (*n* = 3). (G) Images of tumours in each group (*n* = 5). Ctrl: treated with double distilled water; OXA: treated with orexin‐A (0.1 mg/kg/bw; *p < 0.05, **p < 0.01, ***p < 0.001).

To determine whether the suppression of tumour growth caused by orexin‐A is reliant on ferroptosis, we assessed the impact of orexin‐A on the proliferation of GBM cells while using Ferrostatin‐1 (Fer‐1), a ferroptosis inhibitor. Through CCK8, colony formation and Ki67 immunofluorescence assays experiments, we observed that fer‐1 effectively reversed the inhibitory effect of orexin‐A on GBM in vitro (Figure 3A–D). Moreover, when Fer‐1 was combined with orexin‐A, the tumour‐suppressive effects of orexin‐A were mitigated in live animal models (Figure 3F,G).

In summary, orexin‐A induces ferroptosis in GBM cells and this mechanism potentially makes a critical difference in the inhibition of GBM mediated by orexin‐A.

### 3.3 Orexin‐A induces glioma ferroptosis by regulating iron metabolism and inhibiting GPX4

**FIGURE 4:**
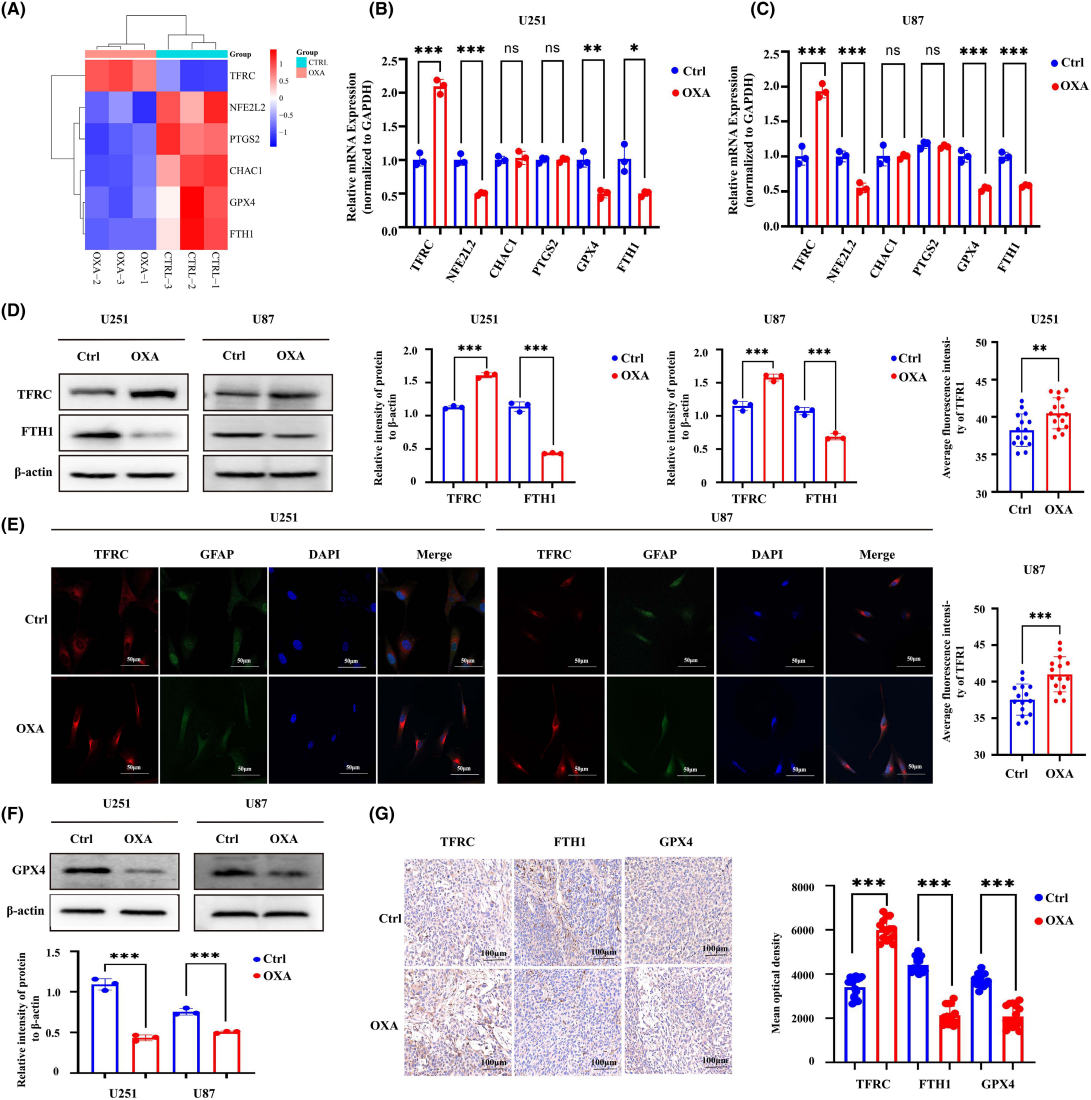
Orexin‐A induces differential expression of ferroptosis marker gene and related protein. (A) The heatmap based on the intersection of DEGs screened by sequencing and Ferroptosis marker genes in FerrDB V2 database. (B and C) The mRNA levels of TFRC, NFE2L2, PTGS2, CHAC1, FTH1 and GPX4 in orexin‐A‐treated U87 and U251 cells were detected by qRT‐PCR. (D) The protein levels of TFRC and FTH1 in orexin‐A‐treated U87 and U251 cells were detected by western blotting. (E) Representative fluorescent images of TFRC in U251 and U87 cells after treated by orexin‐A. Scale bars: 50 μm (400×). (F) The protein levels of GPX4 in orexin‐A‐treated U87 and U251 cells were detected by western blotting. Ctrl: treated with double distilled water; OXA: treated with orexin‐A (0.1 μM). (G) Representative immunohistochemical images of TFRC, FTH1 and GPX4 in Xenograft glioma after treated by orexin‐A. Scale bars: 100 μm (200×). Ctrl: treated with double distilled water; OXA: treated with orexin‐A (0.1 mg/kg/bw; *p < 0.05, **p < 0.01, ***p < 0.001).

To further clarify the mechanism by which orexin‐A induces ferroptosis, the intersection of DEGs screened by sequencing and Ferroptosis marker genes in FerrDB V2 database was used to screen out differentially expressed ferroptosis marker genes and construct heat map. The heatmap based on differentially expressed ferroptosis marker genes revealed that there may be differences in the expression of genes NEF2L2, PTGS2, CHAC1, GPX4 and FTH1 (Figure 4A). Through qRT‐PCR verification, we found that only TFRC, FTH1, NFE2L2 and GPX4 gene expressions were significantly different (Figure 4B,C). TFRC and FTH1 are important regulatory indicators of intracellular iron metabolism, significantly changed expression of which hinted that abnormal iron metabolism may be the underlying mechanism of orexin‐A‐induced ferroptosis.^25^ The results of western blo**t** (WB) and immunohistochemistry (IHC) detection indicated that orexin‐A enhanced the expression of TFRC and decreased the expression of FTH1 in vivo and in vitro (Figure 4D,G). Meanwhile, the immunofluorescence of TFRC further bear out the expression enhancement of TFRC after orexin‐A treatment (Figure 4E). Therefore, orexin‐A may induce ferroptosis by regulating iron metabolism in GBM. At the same time, GPX4 has also been shown by WB and immunohistochemistry (IHC) to be low expressed in GBM treated with orexin‐A (Figure 4F,G). GPX4 is a pivotal regulator of ferroptosis, converts lipid hydroperoxides to lipid alcohols, a process that prevents the formation of iron‐dependent toxic lipid ROS. Inhibition of the function of GPX4 results in the generation of lipid peroxidation and mediates ferroptosis, which may be an important mechanism by which orexin‐A mediates GBM proliferation inhibition.

### 3.4 Orexin‐A targeting NFE2l2 induces ferroptosis in glioma

**FIGURE 5:**
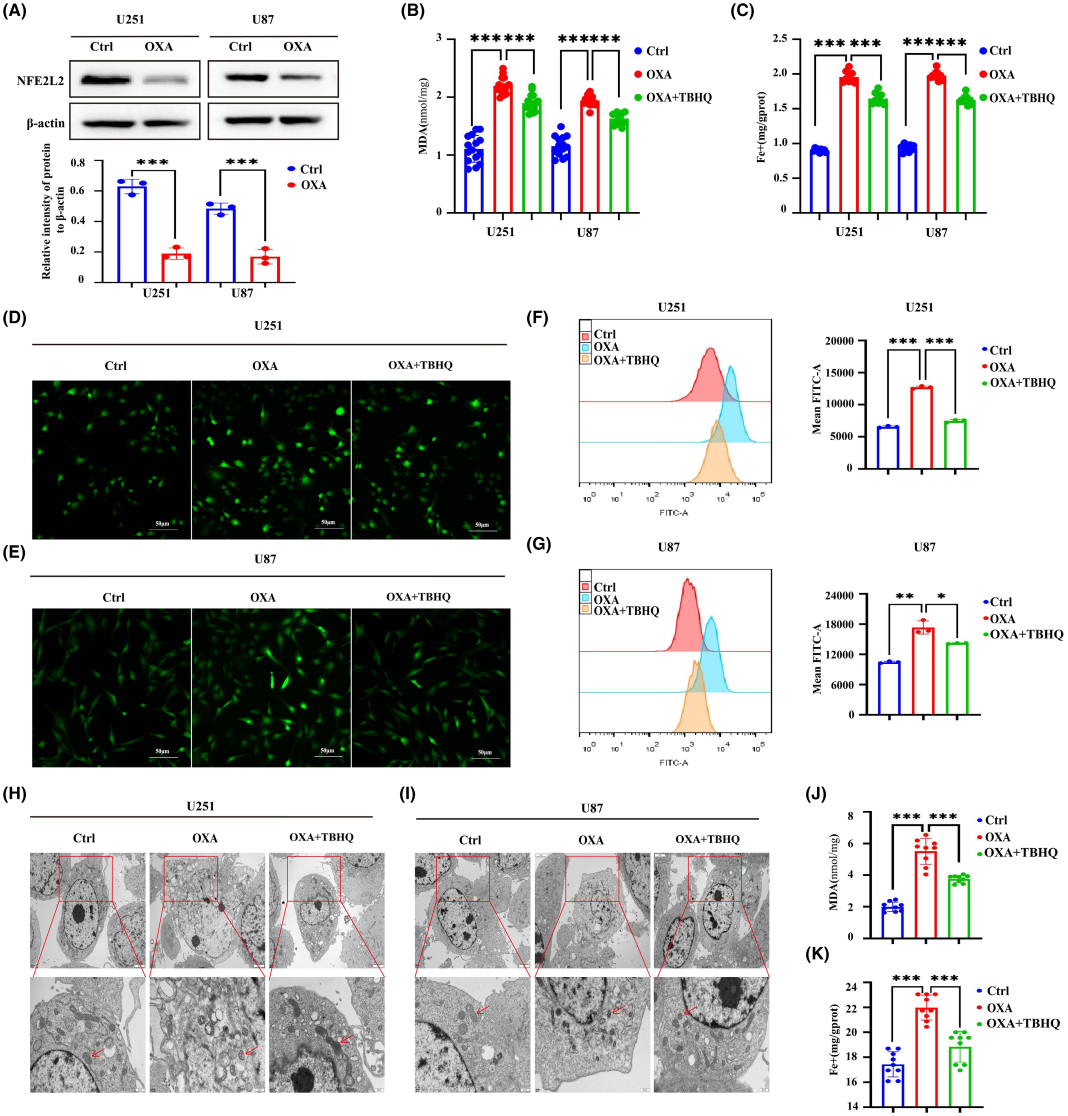
TBHQ‐specific activation of NFE2L2 reverses orexin‐A‐induced ferroptosis. (A) The protein levels of NFE2L2 in orexin‐A‐treated U87 and U251 cells were detected by western blotting. (B) Malondialdehyde (MDA) levels in U251 and U87 cells treated with different reagents for 24 h. (C) The cellular iron concentration in U251 or U87 cells treated with different reagents for 24 h. (D and E) Representative fluorescent images of ROS inU251 or U87 cells treated with different reagents for 24 h. (F and G) Representative flow cytometry analysis ROS in U251 or U87 cells treated with different reagents for 24 h. (H and I) Representative cell and mitochondrial ultrastructural images of U251 or U87 cells treated with different reagents for 24 h. Ctrl: treated with double distilled water; OXA: treated with orexin‐A (0.1 μM); OXA + TBHQ: treated with orexin‐A (0.1 μM) + TBHQ (10 μM). (J) Malondialdehyde (MDA) levels in Xenograft glioma treated with different reagents. (K) The tissue iron concentration in xenograft glioma treated with different reagents. Scale bars: 50 μm (400×). Ctrl: treated with double distilled water; OXA: treated with orexin‐A (0.1 mg/kg/bw); OXA + TBHQ: treated with orexin‐A (0.1 mg/kg/bw) + TBHQ (20 mg/kg/bw; *p < 0.05, **p < 0.01, ***p < 0.001).

**FIGURE 6:**
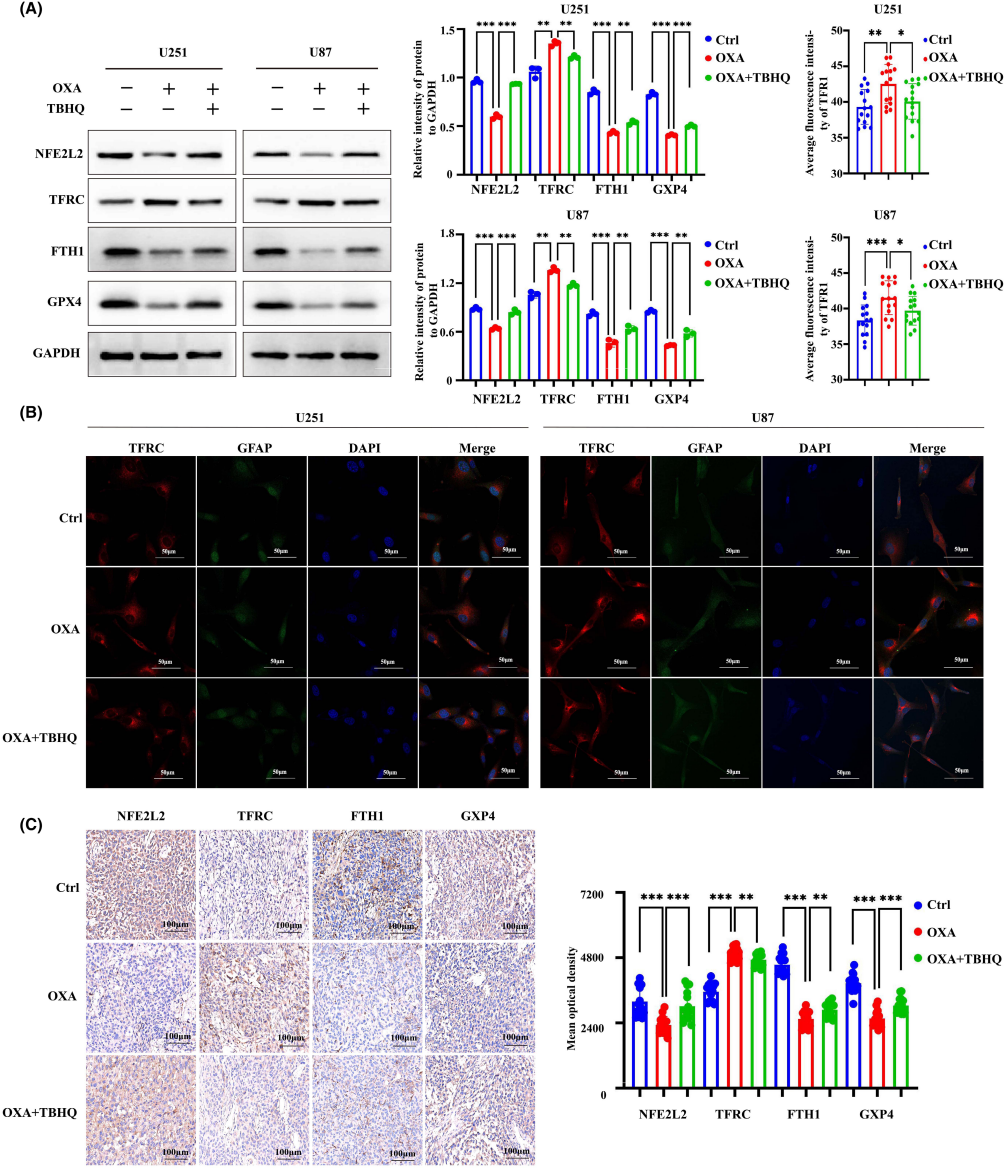
Orexin‐A induces glioblastoma ferroptosis by targeting NFE2L2 by regulating its downstream pathway. (A) The protein levels of NFE2L2, TFRC, FTH1 and GPX4 in orexin‐A‐treated U87 and U251 cells treated with different reagents detected by western blotting. (B) Representative fluorescent images of TFRC in U251 and U87 cells after treated by different reagents. Scale bars: 50 μm (400×) Ctrl: treated with double distilled water; OXA: treated with orexin‐A (0.1 μM); OXA + TBHQ: treated with orexin‐A (0.1 μM) + TBHQ (10 μM). (C) Representative immunohistochemical images of TFRC in U251 and U87 cells after treated by different reagents Scale bars: 100 μm (200×; *p < 0.05, **p < 0.01, ***p < 0.001). Ctrl: treated with double distilled water; OXA: treated with orexin‐A (0.1 mg/kg/bw); OXA + TBHQ: treated with orexin‐A (0.1 mg/kg/bw) + TBHQ (20 mg/kg/bw; *p < 0.05, **p < 0.01, ***p < 0.001).

The WB results demonstrated a notable decrease in the expression of NFE2L2 by orexin‐A (Figure 5A). NFE2L2 serves as a pivotal controller of the intracellular REDOX equilibrium, exerting a vital influence on the regulation of lipid peroxidation and closely associating with the advancement of iron deposition. Based on this, the activation of NFE2L2 was employed to investigate whether orexin‐A modulates ferroptosis in GBM by targeting NFE2L2. Our study found that TBHQ, a NFE2L2 activator, significantly inhibited orexin‐A‐mediated GBM ferroptosis. After pretreatment with TBHQ, orexin‐A‐induced MDA and iron contents increase of GBM cells were significantly reversed (Figure 5B,C,J,K). Moreover, TBHQ mitigated the orexin‐A‐mediated rise in GBM cells ROS levels (Figure 5D–G). Transmission electronic microscopy showed that TBHQ alleviated orexin‐A‐mediated GBM cells vesicle formation, mitochondrial atrophy and ridge disappearance (Figure 5H,I). In addition, further molecular mechanism studies found that activating NFE2L2 enhanced the expression of FTH1 and GPX4 and inhibited the expression of TFRC in GBM cell lines (Figure 6A). Immunofluorescence of TFRC further confirmed that TBHQ reduced TFRC expression on the surface of GBM cell lines (Figure 6B). Activation of NFE2L2 in mouse xenograft tumour models also enhanced the expression of FTH1 and GPX4 and decreased the expression of TFRC to some extent (Figure 6C). Therefore, orexin‐A may modulate the expression of TFRC, FTH1 and GPX4 downstream by targeting NFE2L2, thereby inducing GBM ferroptosis both in vivo and in vitro.

## 4 DISCUSSION

Ferroptosis is one of the patterns of PCD that has aroused much concern in recent years, which can affect the development and prognosis of many tumours.^26^ GBM represents a prevalent form of primary malignant tumours in the CNS, but limited treatment options and unsatisfactory treatment results still make the treatment of glioma a great challenge.^27^ Ferroptosis, as the most abundant PCD process in glioma,^24^ affects tumour cell proliferation, tumour necrosis, angiogenesis and immune resistance,^28^ which makes a critical difference in the progression of glioma. Induction of ferroptosis has become an appealing tactics for addressing GBM.

Orexin‐A induces proliferation inhibition of GBM both in vivo and in vitro. Pathway enrichment analysis for the multi‐omics sequencing data from orexin‐A‐treated GBM cell lines revealed a significant enrichment of ferroptosis. Subsequent detection of ferroptosis indicators both in vitro and in vivo experiments confirmed that orexin‐A triggers ferroptosis in GBM. The reversal of orexin‐A‐induced GBM growth inhibition by Ferristatin‐1 suggests that ferroptosis is the primary mechanism behind orexin‐A‐induced GBM growth inhibition. Previous studies have suggested that orexin‐A plays an antitumour role mainly by inducing apoptosis.^12^, ^18^, ^19^ Our study is the first to identify the potential of orexin‐A to induce ferroptosis in tumours, which further enriches the antitumour pathway of orexin‐A. Although ferroptosis and apoptosis differ in terms of cell morphology and biochemical characteristics, there is increasing evidence supporting their synergistic or complementary interaction.^29^ Under certain conditions, apoptosis can transition into ferroptosis, and ferroptosis can enhance cell susceptibility to apoptosis, depending on the cellular environment and death triggers.^30^, ^31^ Therefore, the antitumour effect induced by orexin‐A may be the combined result of ferroptosis and apoptosis, but the proportion of ferroptosis and apoptosis is different in different tumour backgrounds. In addition, the combination of drugs targeting both mitochondrial apoptosis and ferroptosis is a promising new approach for GBM treatment.^32^, ^33^ Orexin‐A shows significant ferroptosis inducing potential in glioma, but whether it can induce apoptosis needs further study. But in any case, as a potential inducer of ferroptosis in GBM, orexin‐A is a promising antitumor drug.

Intracellular iron accumulation is one of two central biochemical events leading to ferroptosis.^34^ Iron metabolism within cells is strictly regulated, including iron intake, storage and excretion.^35^ The disruption in iron metabolism is a significant factor in the increase of intracellular unstable iron pool, which ultimately leads to ferroptosis.^36^ Several genes responsible for regulating iron metabolism have been identified as making a critical difference in the induction of ferroptosis. One such gene is TFRC, which encodes transferrin receptor 1, a critical iron transporter located on the cellular membrane. In fact, the expression levels of TFRC are directly correlated with the cellular iron load. The TFRC transports iron ions through a process of endocytosis, where they are reduced to iron ions and then transported into the cytoplasm, forming the unstable iron pool (LIP).^37^ Increased expression of TFRC has been linked to increased susceptibility to ferroptosis and has even been shown to induce ferroptosis in various disease models.^38^, ^39^, ^40^ Ferritin, a protein responsible for storing iron within cells, is composed of 24 subunits comprising of two variations: ferritin heavy chain (FTH) and ferritin light chain (FTL). These subunits form the molecular basis for ferritin to function as an iron storage protein and make a critical difference in maintaining iron homeostasis.^41^ Expression defects in FTH and FTL can significantly increase the presence of unstable iron in cells and promote the occurrence of ferroptosis.^36^, ^42^ FPN (SLC40A1), a member of the large solute carrier gene family, is a transmembrane protein mainly expressed in macrophages, duodenum and hepatocytes. It is thought to be the only (or major) exporter of iron, although its structure and the molecular mechanism of iron output are not fully understood, the absence of FPN has also been shown to be involved in the occurrence of ferroptosis.^43^, ^44^ In our study, we observed an increase in TFRC expression and a decrease in FTH1 expression in gliomas following orexin‐A treatment. In GBM, metabolic reprogramming in the tumour microenvironment leads to high expression of TFRC, which provides more iron for tumour progression and drives tumour progression.^45^ However, the high expression of TFRC also results in elevated iron levels in GBM, increasing its sensitivity to ferroptosis. Additionally, the decreased expression of FTH1 induced by orexin‐A leads to the deficiency of iron storage function, which increases the unstable iron in gliomas, providing a crucial foundation for the induction of ferroptosis.

Ferroptosis is a type of cellular demise resulting from uncontrolled membrane lipid peroxidation (LPO).^46^ Normally, LPO is regulated through metabolic homeostasis, only occurring when indispensable constraints are met and the anti‐peroxidant defence system fails. The activity of the selenoperoxidase Glutathione Peroxidase 4 (GPX4) is the cornerstone of the anti‐peroxidant defence. Genetic studies conducted in cells and mice have identified the GPX4 as a pivotal regulator of ferroptosis.^47^ The enzyme GPX4 utilizes glutathione (GSH) to convert lipid hydroperoxides into lipid alcohols, effectively preventing the creation of iron‐dependent toxic lipid ROS and making a critical difference in suppressing ferroptosis.^48^ Impairment of GPX4's functionality caused lipid peroxidation and can induce ferroptosis.^49^ Furthermore, GPX4‐mediated anti‐peroxidation also makes a critical difference in GBM.^50^, ^51^ There is mounting evidence supporting the strategy of targeting GPX4 to induce iron‐dependent cell death as a promising approach to treat gliomas.^5^, ^52^ In our research, we observed that orexin‐A reduces the expression of GPX4 in GBM, and the increase in MDA and ROS further demonstrates that orexin‐A induces lipid peroxidation‐mediated ferroptosis in GBM by inhibiting GPX4.

NFE2L2 (NF‐E2‐related factor 2) is a well‐known oxidative stress response transcription factor that significantly contributes to the oxidative stress defence mechanism. Accumulated evidence that NFE2L2 mediates the fine regulation of ferroptosis.^53^ By modulating key pathways involved in ferroptosis, NFE2L2 acts as an inhibitor of this iron‐dependent form of cell death.^54^ It accomplishes this by either activating or suppressing the expression of genes involved in intracellular labile iron metabolism,^36^ the GSH‐GPX4 pathway^55^ and the FSP1‐CoQ pathway.^56^ Furthermore, NFE2L2 also controls ferroptosis by regulating lipid metabolism and cell differentiation. This multifaceted regulation of ferroptosis by NFE2L2 is believed to have been obtained during the evolution of multicellular organisms, allowing ferroptosis to be used to maintain homeostasis, including cancer suppression.^56^ Targeted modulation of NFE2L2 and its downstream genes in gliomas has been shown to induce ferroptosis.^57^ In our study, we observed that orexin‐A can induce decreased expression of NFE2L2, and TBHQ activation of NFE2L2 can significantly reverse orexin‐A‐mediated ferroptosis in GBM. This activation also leads to increased expression of GPX4, FTH1 and decreased expression of TFRC. Consequently, orexin‐A may induce ferroptosis in gliomas by selectively targeting NFE2L2 and regulating the downstream expression of GPX4, TFRC and FTH1.

In conclusion, we confirmed in this study that ferroptosis is a pathway leading to orexin‐A‐induced GBM proliferation inhibition. Orexin‐A regulates the expression of TFRC, FTH1 and GPX4 by targeting oxidative stress response transcription factor NFE2L2.The accumulation of unstable iron and the depletion of GPX4 play crucial roles in orexin‐A‐induced ferroptosis in GBM.

## AUTHOR CONTRIBUTIONS

**Rengzheng Huan:** Data curation (equal); investigation (equal); visualization (equal); writing – original draft (equal). **Jiqin Zhang:** Data curation (equal); investigation (equal); writing – original draft (equal). **Jianhe Yue:** Software (equal); validation (equal); visualization (equal). **Sha Yang:** Software (equal); validation (equal); visualization (equal). **Guoqiang Han:** Project administration (equal); resources (equal); supervision (equal). **Yuan Cheng:** Conceptualization (equal); methodology (equal); writing – review and editing (equal). **Ying Tan:** Conceptualization (equal); funding acquisition (equal); methodology (equal).

## CONFLICT OF INTEREST STATEMENT

There are no conflicts of interest in this study.

## Supporting information


Table S1.

## Data Availability

The data that support the findings of this study are available from the corresponding author upon reasonable request.
