# Supplementary material for: Orexin‐A mediates glioblastoma proliferation inhibition by increasing ferroptosis triggered by unstable iron pools and GPX4 depletion
Source: J Cell Mol Med. 2024 Apr 29;28(9):e18318. doi: 10.1111/jcmm.18318 (PMC11058333; doi:10.1111/jcmm.18318)
Supplement: Supplementary file 1 — Table S1. [file JCMM-28-e18318-s001.docx]

**Table S1: List of primers for differential genes associated with ferroptosis**

TFR1 F GGCTACTTGGGCTATTGTAAAGG

TFR1 R CAGTTTCTCCGACAACTTTCTCT

FTH1 F CCCCCATTTGTGTGACTTCAT

FTH1 R GCCCGAGGCTTAGCTTTCATT

GPX4 F GAGGCAAGACCGAAGTAAACTAC

GPX4 R CCGAACTGGTTACACGGGAA

PTGS2 F CTGGCGCTCAGCCATACAG

PTGS2 R CGCACTTATACTGGTCAAATCCC

NFE2L2 F TCAGCGACGGAAAGAGTATGA

NFE2L2 R CCACTGGTTTCTGACTGGATGT

GAPDH F GGAGCGAGATCCCTCCAAAAT

GAPDH R GGCTGTTGTCATACTTCTCATGG

CHAC1 F GAACCCTGGTTACCTGGGC

CHAC1 R CGCAGCAAGTATTCAAGGTTGT
